# Analysis, Properties, and Applications of Insect-Derived Chitosan: A Sustainable Path to Functional Polysaccharide Materials

**DOI:** 10.3390/gels11040291

**Published:** 2025-04-15

**Authors:** Hanna L. Schäfer, Lars Gandras, Laura Schneider, Marco Witthohn, Kerstin Troidl, Kai Muffler, Clemens K. Weiss

**Affiliations:** 1 Life Sciences and Technology, University of Applied Sciences Bingen, Berlinstrasse 109, 55411 Bingen, Germany; 2 Department of Vascular and Endovascular Surgery, Cardiovascular Surgery Clinic, University Hospital Frankfurt, 60590 Frankfurt, Germany; 3 Department of Pharmacology, Max Planck Institute for Heart and Lung Research, 61231 Bad Nauheim, Germany; 4 Analytical Core Facility, University of Applied Sciences Bingen, Berlinstrasse 109, 55411 Bingen, Germany

**Keywords:** insect farming, polysaccharide, biopolymer, chitosan, chitin, functional material, hydrogel, analytical techniques

## Abstract

Chitin and its deacetylated derivative, chitosan, are biopolymers of significant interest due to their biocompatibility, biodegradability, and wide-ranging applications in biomedical, environmental, and industrial fields. The exploitation of crustaceans as the traditional source of chitosan raises concerns about overfishing and ecological sustainability. Modern insect farming, in contrast, offers advantages such as a circular insect-based economy leading to a reduced carbon footprint. This review explores the potential of insect-derived chitosan as an alternative, emphasizing its environmental benefits during production, functional properties, and potential applications. Several aspects of key analytical techniques for chitin and chitosan characterization, including photometric, chromatographic, and spectroscopic methods, are also discussed. The review underscores the versatility of insect-derived chitosan in biomedical applications, including wound healing and drug delivery, as well as its potential in agriculture, packaging, and wastewater treatment.

## 1. Introduction

Chitosan and its precursor chitin have emerged as biopolymers of considerable interest due to their remarkable properties and diverse applications across various sectors, particularly in material science and biotechnology. Chitin, predominantly sourced from crustacean exoskeletons, is a naturally occurring polysaccharide composed of *N*-acetylglucosamine units linked by *β*-(1,4)-glycosidic bonds. Upon deacetylation, chitin is converted into chitosan, characterized by amino groups that bestow functional properties advantageous for numerous applications [1,2,3]. The biocompatibility and biodegradability of chitosan position it as an ideal candidate for biomedical applications, including drug delivery systems and wound dressings, where its capability to encapsulate therapeutic agents and facilitate healing is invaluable [4]. In environmental science, chitosan has gained prominence as an effective biosorbent for the removal of heavy metals and organic pollutants from wastewater, supporting sustainable water management practices [5]. However, the traditional sourcing of chitosan from crustaceans raises significant environmental concerns. The crustacean industry is linked to habitat destruction through practices such as bottom trawling, which severely impact benthic environments. The Food and Agriculture Organization (FAO) reports [6] that overfishing and destructive fishing practices have resulted in the depletion of many global fish stocks, particularly in ecologically sensitive regions like the Mediterranean Sea [7].

In contrast, there is an increasing interest in alternative sources of chitosan, particularly from insects, which offer more sustainable options. Insect farming requires significantly fewer resources, such as land and water, and demonstrates greater efficiency in feed conversion. Additionally, insects play a crucial role in waste reduction and recycling, enabling the conversion of organic waste into valuable protein sources [8,9]. This shift towards insect-based chitosan production not only addresses sustainability concerns but also supports eco-friendly practices in biopolymer production [10].

Species such as *Hermetia illucens* (black soldier fly) are recognized for their ecological and economic advantages over traditional crustacean sources. Insect farming consumes significantly less land, water, and feed while generating lower greenhouse gas emissions [4,11]. This strategy mitigates the environmental burden linked to chitin and chitosan extraction from marine ecosystems, which are already threatened by overfishing and pollution [12]. Moreover, insect farming presents a scalable and efficient solution by integrating waste management systems that convert organic waste into high-value insect biomass [13,14].

The concentration of chitin and chitosan in insect larvae is significantly influenced by rearing conditions, including the larval diet and environment. Research indicates that variations in feeding regimes—such as the type and quality of substrates—can lead to substantial differences in chitin accumulation within the larvae [9,15]. This adaptability enhances the nutritional profile of the larvae and optimizes chitin yield, rendering it a more efficient source compared to traditional crustacean methods. Additionally, the capacity to control these parameters allows for the fine-tuning of chitin production processes, potentially resulting in improved chitosan properties that are desirable for various applications in material science and biotechnology [16]. Thus, the exploration of optimal rearing strategies for insects presents a promising avenue for enhancing chitin yields while simultaneously promoting environmental sustainability.

Extracting chitosan from insect sources provides numerous functional advantages that expand its applicability across various fields. Research indicates that chitosan derived from insects demonstrates comparable, if not superior, properties concerning biocompatibility, antimicrobial activity, and biodegradability—critical attributes for medical applications such as drug delivery systems and wound healing [17,18,19]. The superior antimicrobial properties of insect-derived chitosan make it particularly valuable in biomedical contexts, effectively preventing infections and promoting tissue regeneration.

Furthermore, the valorization of insect biomass for both chitosan and protein enhances sustainability across sectors such as food production, pharmaceuticals, and agriculture, while fostering economic growth by creating new markets and job opportunities [9,20,21]. Chitosan has been shown to enhance plant growth and stimulate defense mechanisms against pathogens, acting as a biopesticide that induces systemic resistance in plants, thereby improving resilience to diseases [22,23]. The incorporation of insect-derived chitin and chitosan into agricultural practices represents an innovative approach to enhance sustainability, improve crop yields, and promote environmental health.

**Figure 1:**
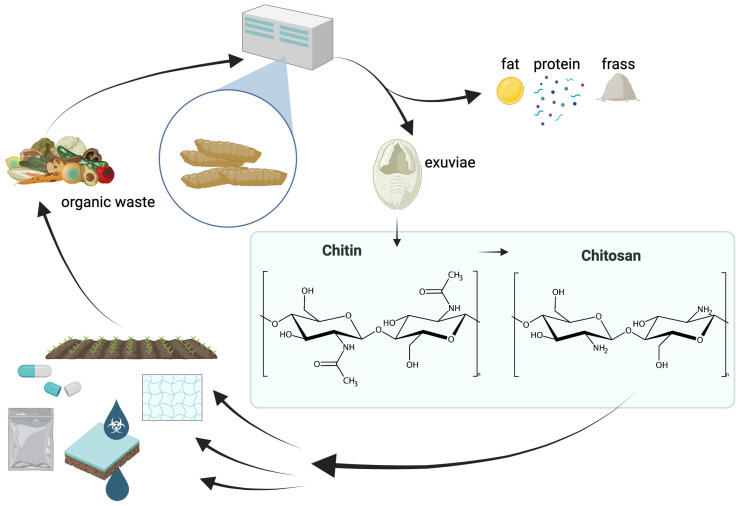
Chitin and chitosan are, in addition to insect-derived fat and protein, valuable bioresources suitable for various applications ranging from packaging to biomedical applications. Being biodegradable, chitosan-based materials can be regarded as organic waste, which is a potential feedstock for insect farming. (created with BioRender).

Insect farming, with its lower environmental impact compared to traditional sources, supports circular economy principles by recycling organic waste into high-value products (Figure 1). While further research is warranted to optimize extraction methods and enhance the functionality of insect-derived chitosan, the potential for its industrial applications in material science and biotechnology is vast [5,16]. This multifaceted approach contributes to both ecological sustainability and economic resilience, positioning insect-derived chitosan as a key player in the future of biopolymer development.

**Figure 2:**
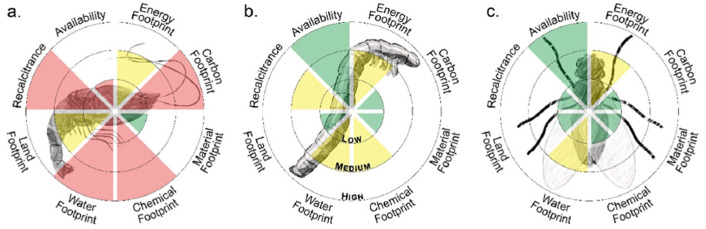
The overall qualitative assessment of the sustainability of shrimps (**a**), mealworms (**b**), and larval exoskeletons (**c**) as sources of nanochitin includes various ecological and production aspects. Green indicates beneficial, yellow medium, red less beneficial impact on the assessed aspects. Reprinted with permission from Eva Pasquier et al. [24], ACS Sustainable Chem. Eng. 2021, 9, 13618–13629. Copyright 2021 American Chemical Society.

A comparative study [24] on the impact of several key ecological aspects such as availability, energy, carbon, material, chemical, water, and land footprint and recalcitrance. Figure 2 shows the results, which clearly show the benefits of insects as a chitin source in terms of availability and ecological footprint, especially in considering the carbon, water, and land impacts.

## 2. Chitin and Chitosan—Sources, Properties and Extraction Methods

### 2.1. Occurrence and Structure of Chitin

**Figure 3:**
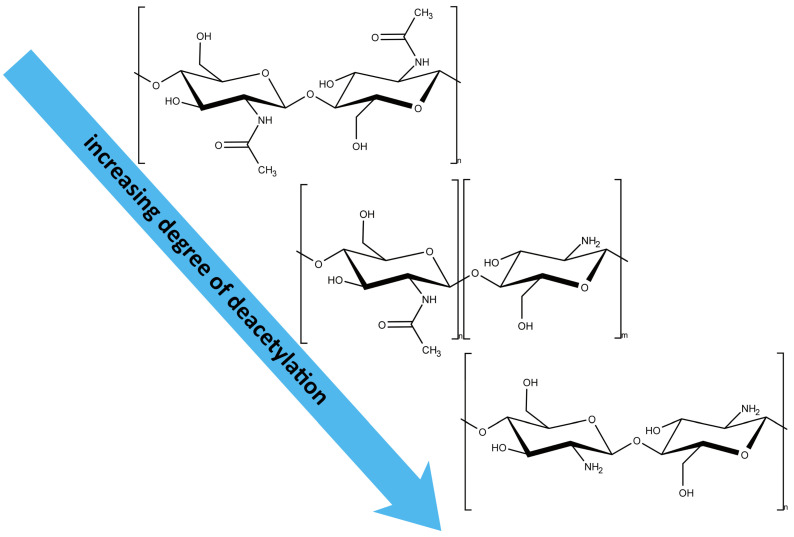
Chitin (**top**), partially deacetylated chitin (**middle**), and deacetylated chitin (**bottom**). Chitin with a degree of deacetylation of higher than 50% is typically referred to as chitosan in the literature.

Chitin is a naturally occurring polysaccharide and, after cellulose, is the second most abundant biopolymer on Earth. Its widespread presence in crustacean exoskeletons, insect cuticles, and fungal cell walls results in an estimated biospheric production of approximately 10^11^ tons annually [25]. Chitin’s structure consists primarily of chains of *N*-acetyl-D-glucosamine units (see Figure 3).

**Figure 4:**
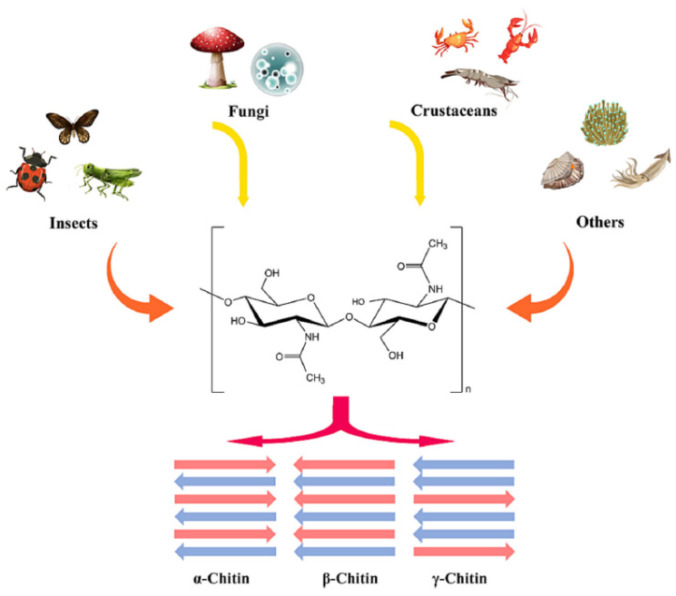
Sources and structure of chitin. Reprinted from Jiran Lv et al. [26], Chitin and chitin-based biomaterials: A review of advances in processing and food applications, Carbohydrate Polymers, 299, 120142, Copyright (2023), with permission from Elsevier.

The arrangement of the polymeric chains gives rise to three main structural forms, as shown in Figure 4: *α*-chitin, *β*-chitin, and *γ*-chitin. These variations are a result of differences in chain alignment and the biological source. The most abundant form, *α*-chitin, characterized by an anti-parallel arrangement of molecular chains, is common in arthropods and crustaceans. *β*-chitin, which has parallel chains, is found in sea diatoms or squid pens, whereas *γ*-chitin is a form combining both arrangements. This form is found in fungi and yeasts.

Despite its abundance, chitin’s compact structure and high hydrophobicity make it nearly insoluble in water and organic solvents, significantly limiting its applications [27,28]. Consequently, direct uses of chitin are primarily confined to fields like tissue engineering and agriculture, where solubility is less critical. In most other applications, chitin has to be deacetylated to produce chitosan, a water-soluble derivative (see Figure 3). The physical and chemical properties of both chitin and chitosan depend greatly on their source and the methods used for extraction and purification [29].

### 2.2. Sources and Composition of Chitin

The principal sources of chitin are arthropods and fungi [26] (see Figure 4). Within crustaceans, chitin is a structural element of the exoskeleton, comprising 15–40% of its mass. The demand for chitin from crustaceans has spurred interest in insect-derived chitin, particularly with the rise of insect farming as a sustainable protein source. Insect exoskeletons generally contain over 30% chitin by mass, making them a viable alternative.

Crab and shrimp exoskeletons are composed of 30–40% protein, 30–50% calcium carbonate (CaCO_3_), and 20–30% chitin (all numbers given as wt%), along with smaller amounts of pigments, other minerals, and lipids [12,26]. In contrast, fungi, the second-largest source of chitin, contain 1–15% chitin within their cell walls, and some also contain chitosan [25]. Insects exhibit a unique composition, with biomass consisting of 30–60% protein, 10–25% lipid, 5–30% chitin, 5–10% catechins, and 2–10% mineral content, including calcium, phosphorus, potassium, and magnesium salts [25,30]. This structural matrix necessitates purification steps to remove non-chitinous components.

### 2.3. Purification of Chitin

Purifying raw chitin to produce chitosan involves either chemical or biotechnological extraction methods. Both routes involve removal of inorganic compounds, deproteinization, defatting, and, depending on the composition, deodorization and decoloration, removing melanin [7,13,14,25,26,31,32,33]. The presence of melanin in insects is a major difference from crustaceans. This has to be considered during workup procedures and also when considering possible applications.

**Figure 5:**
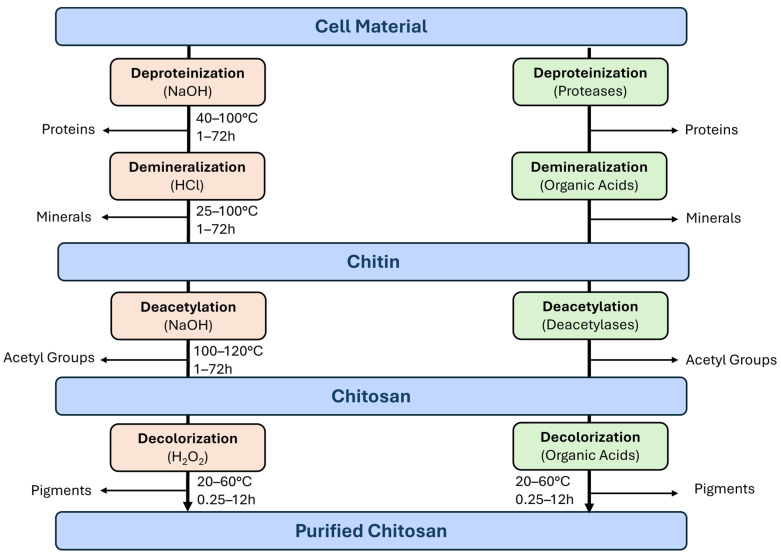
Biological and chemical methods for chitin extraction. The boxes with orange shade depict the steps of the chemical method, the boxes shaded in green depict the purification steps of the biotechnological method.

Typical chemical purification begins with demineralization, where chitin is treated with hydrochloric or acetic acid to dissolve salts. Acidic demineralization may also reduce the degree of polymerization, thereby affecting chitosan’s solubility and reducing the viscosity of a solution. Next, deproteinization occurs with sodium hydroxide to remove proteins, often requiring 24–48 h at temperatures between 60 and 90 °C. Conditions vary depending on the source, as crab chitin typically has a higher salt content, while insect-derived chitin generally contains more lipids. For color-sensitive applications, bleaching agents like hydrogen peroxide, sodium hypochlorite, or acetone are sometimes used (see Figure 5). Extended alkaline treatment following demineralization and deproteinization can transform chitin into chitosan through deacetylation. Structural analysis of chitosan typically requires multiple analytical techniques, as detailed in Section 3.

Chemical purification, however, is labor intensive, energy consuming, and produces waste streams requiring neutralization, purification, and disposal. Biological extraction, an alternative approach, utilizes enzymes (mainly proteases) or entire microorganisms to break down proteins and other components. Although environmentally preferable, this method generally yields lower chitin content [14,26]. Both chemical and biological methods require pre-treatment steps, including washing with water and detergents, followed by drying and grinding to attain an appropriate particle size distribution [34].

Since neither method has proved completely effective, novel techniques are under development, such as the use of ionic liquids [35,36,37], deep eutectic solvents [38,39], supercritical fluid extraction, electrochemical processes, and ultrasound-assisted extraction [26].

### 2.4. Conversion of Chitin to Chitosan: Chemical and Biotechnological Paths

The conversion of chitin to chitosan typically involves the removal of acetyl groups (deacetylation) to increase its solubility and bioavailability. The degree of deacetylation (DA) significantly influences the functional properties of chitosan, making precise control essential for tailored applications [40].

Chemical deacetylation is the most common method for converting chitin to chitosan. It typically involves treating chitin with concentrated alkali solutions (e.g., NaOH) at elevated temperatures (80–120 °C). The degree of deacetylation depends on factors such as alkali concentration, reaction time, and temperature. Optimal conditions (e.g., 50% NaOH at 120 °C for 3–12 h) can yield high-molecular-weight chitosan with well-controlled DA. However, chemical methods present challenges, including environmental hazards due to the use of concentrated alkalis [41] and difficulties in controlling polymer chain length (degree of polymerization, DP). High temperatures can lead to depolymerization, reducing the molecular weight and functional properties of chitosan. Chemical depolymerization involves breaking glycosidic bonds in chitosan to produce shorter oligomers. Common methods include acid hydrolysis with HCl and oxidative depolymerization using H_2_O_2_ [40]. While effective, these methods often result in undefined oligomer mixtures and low yields, limiting their utility for producing highly defined chitosan oligomers [42]. In the biotechnological conversion process of chitin to chitosan, chitin deacetylases (CDAs) catalyze the hydrolysis of acetyl groups, offering a more environmentally friendly alternative to chemical methods. CDAs, derived from fungi, bacteria, and marine organisms, produce chitosan and partially acetylated oligomers with well-defined DA and acetylation patterns (P_A_) [43]. For example, CDAs from *Mucor rouxii* and *Colletotrichum lindemuthianum* have demonstrated distinct catalytic mechanisms, enabling targeted deacetylation [1]. Enzymatic deacetylation is highly specific, allowing precise control over product properties. However, the high cost of enzymes and slower reaction rates compared to chemical methods remain significant challenges [40]. Chitinolytic enzymes, such as chitinases and chitosanases, break down chitin and chitosan into shorter oligomers. These enzymes offer high specificity, enabling the production of defined oligomers with desired DP and DA [44]. For instance, bacterial chitosanases from *Bacillus subtilis* have been used to produce chitosan oligomers with DP1–DP6 [45]. Combining chitinases with CDAs can further enhance process specificity, producing oligomers with novel structures and functionalities.

Combining chemical and enzymatic methods can leverage the strengths of both approaches. For instance, chemical deacetylation can produce partially deacetylated chitin, which is subsequently depolymerized using chitosanases. This approach reduces chemical usage while achieving higher specificity in product characteristics. Synthetic biology also offers promising ways for sustainable chitosan production. For example, engineered microbes expressing chitin synthase and chitin deacetylase genes can biosynthesize defined chitosan oligomers (COS) directly from glucose. While still in its infancy, this approach could bypass traditional extraction and conversion processes. However, since crustacean shell chitin is an abundant waste material, its processing is more sensible and sustainable than full COS synthesis from glucose.

### 2.5. Insect-Based Chitin as a Sustainable Alternative

Insect-derived chitin is increasingly recognized as a sustainable alternative to traditional marine-based sources. The fundamental chemical structure of chitin from various sources remains the same: it is a polysaccharide consisting of *N*-acetyl-D-glucosamine units connected by *β*-(1,4)-glycosidic bonds.

Compared to marine-based chitin, insect chitin exhibits some notable chemical and structural differences. Insect exoskeletons have a lower inorganic content, especially in calcium carbonate, making demineralization more straightforward and environmentally friendly. This lower mineral content not only reduces the energy requirements but also minimizes the need for chemical reagents, resulting in a more sustainable production process.

## 3. Analysis of Chitin and Chitosan

The crystal structure of chitin is typically assessed with X-ray diffraction methods, and the morphology with electron microscopy [13,46,47]. Here we focus on chemical analyses for qualitatively and quantitatively analyzing chitin and chitosan and on methods for the determination of the molecular weight and the degree of acetylation/deacetylation.

Although the methods are applicable to chitin and chitosan from each source, several species-specific features have to be considered, such as the presence of pigments (melanin), potentially interfering with a given method.

### 3.1. Methods for Quantification

For the various applications of chitin and chitosan in a wide range of areas, it is of great importance to analyze the samples and determine their yield and purity. Reliable and reproducible methods are therefore essential to enable further processing.

Current research is focused on the investigation of chitin and chitosan obtained from all sources. This part of the review is intended to present the various analytical methods for the quantitative determination of chitin or chitosan and provide an overview of the current state of research in this field.

#### 3.1.1. Photometric Methods

**Figure 6:**
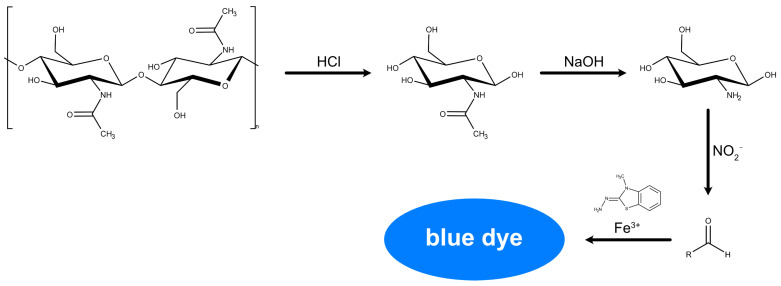
Reaction sequence employed in the method of Lehman and White.

With more than 100 years of development [48,49,50,51,52,53,54,55,56,57,58,59,60,61,62,63,64], photometric methods are still used for the chemical analysis of chitin and chitosan. Despite the availability of more advanced methods, these methods provide an easy and convenient way to quantify chitosan. Most frequently used today is the method of Lehman and White [65], which builds on the earlier work of Sawicki [60] and Tsuji [66]. It is based on depolymerization of chitin into monomer units by acid hydrolysis. *N*-acetyl-D-glucosamine is subsequently converted into an aldose and eventually a blue dye by treatment with sodium nitrite, ammonium sulfamate, MBTH, and FeCl_3_. The reaction sequence is shown in Figure 6.

This method has inspired various adaptations, improving selectivity [67] and reducing reaction times [68]. The methods have been applied to various biological sources, underlining the potential of photometric methods. In addition, other methods have also been further developed and refined. The combination of photometry with ion-exchange chromatography [69,70,71,72,73] allows quantification of *N*-glucosamine by conversion to the dye and the quantification of *N*-acetyl-glucosamine with ion-exchange chromatography [73]. MBTH-based and other photometric methods have been used for the quantification of chitin in fungi [74,75,76,77,78,79,80,81], crustaceans [82,83,84], and insects [85,86,87,88].

#### 3.1.2. Methods Based on Fluorescence

In addition to photometric methods, methods based on fluorescence have recently emerged for the quantitative analysis of chitin. Costa-de-Oliveira used flow cytometry for determining the chitin content in fungal walls. For this purpose, the cells used are stained with Calcofluor White, and the emission is measured with a flow cytometer. The chitin content is determined by calculating the staining index. The fluorescence of Calcofluor White, which specifically binds to chitin, can also be used to localize chitin [89,90] or quantify [91] the amount of chitin. Flaven-Pouchon and Moussian [90] investigated chitin staining in whole preparations of *Drosophila melanogaster* using the fluorescence brightener 28 at elevated temperature (65 °C) for improved dye permeation. The samples were analyzed with fluorescence microscopy for chitin localization, without quantification. Henriques et al. [91] also employ Calcofluor White and compare the quantification results obtained with a microplate reader to the results of the Lehman and White method. Another approach by Maeda et al. [92] used fluorescence from metal chelates formed by a reaction of Zn(II) and *N*-pyridoxylidene.

#### 3.1.3. Chromatographic Methods

The most common methods for chitin or saccharide analysis are based on photometry due to its simplicity and cost-effectiveness, as it does not require expensive equipment. In contrast, chromatographic methods, such as gas chromatography (GC), liquid chromatography (LC, HPLC, UPLC), and ion exchange chromatography (IEC), often require labor- and time-intensive derivatization steps.

GC has been utilized by Radhakrishnamurthy et al. [93] and Hase and Matsushima [94]. Both strategies increase the volatility of saccharides by silylation. Bierstedt et al. [95] compare the results of the photometric Lehman and White method to a method based on pyrolysis gas chromatography, coupled with mass spectrometry (Py-GC-MS). The photometric method yields reliable quantification of chitinous compounds in modern and decayed arthropods, while the analysis of fossil arthropods requires detailed molecular information, only obtained by Py-GC-MS.

Recent efforts on HPLC-based methods include the work of Ekblad and Näsholm [96], Zhu et al. [97], Crespo et al. [98], Lopez-Cervantes et al. [99], M. Šulc [100], and Zhou et al. [101]. Lopes-Cervantes [99] uses hydrochloric acid for hydrolysis of chitin followed by pre-column derivatization with 9-fluorenylmethylchloroformate (FMOC-Cl), enabling UV detection after HPLC separation. Crespo et al. [98] use RI and MS detection after separating a hydrolyzed sample with HPLC, avoiding the necessity of glucosamine derivatization. Ekbald and Näsholm [96] as well as Zhou et al. [101] use the fluorescent properties of FMOC for HPLC detection, with FMOC-Cl or FMOC-Su for derivatization, after removal of interfering components such as proteins or amino acids. Šulc further refined the process with defatting, deproteinization, and hydrolysis prior to derivatization with FMOC-Su and HPLC analysis.

Han and Heinonen [102] describe an adapted and optimized method for the use of UPLC with fluorescence detection. Their method not only proved effective for chitin analysis but also enhanced accuracy in determining both protein and fiber content.

### 3.2. Determination of Degree of Deacetylation

While photometric and chromatographic methods allow quantification of chitin and its derivatives, another critical aspect of chitin and chitosan analysis is the determination of the degree of deacetylation (DD). This parameter significantly influences the material’s physicochemical [103], chemical, and biological properties, making its accurate measurement essential. As mentioned above, poly-*N*-acetyl-D-glucosamine with DD < 50% is typically referred to as chitosan, with DD > 50% as chitin.

It has to be borne in mind that solution-based methods, such as colloidal titration or solution NMR, only determine the DD of completely dissolved chitosan. Thus, measurements on insoluble or partially dissolved chitosan have to be evaluated carefully. As the solubility is one of the parameters depending on the DD [103,104], this has to be considered when dealing with the results of DD measurements.

Thus, despite several shortcomings, discussed below, IR spectroscopy has frequently been used for the determination of the DD from solid chitin or chitosan. Several measurement techniques, including transmission spectroscopy on neat samples, in KBr pellets, attenuated total reflection (ATR), or diffuse reflectance infrared Fourier transform spectroscopy (DRIFTS) have been applied and compared [105].

Several characteristic vibrational modes have been identified and assigned to functional groups associated with chitin and chitosan [105,106]. Three notable equations relating the absorbance of two distinct vibration bands have widely been used in the literature:

Moore and Roberts [107]: $DA=\frac{A_{1655}}{A_{3450}}\cdot \frac{100}{1,33}$

Baxter et al. [108]: $DA=\frac{A_{1655}}{A_{3450}}\cdot 115$

Brugnerotto et al. [105]: $\frac{A_{1320}}{A_{1420}}=0.3822+0.03133DA$

While the equations presented by Moore [107] and Baxter [108] relate the absorbance of the amide I band ($\tilde{\nu }=1655\;{cm}^{-1}$) to the broad OH-stretching band ($\tilde{\nu }=3450\;{cm}^{-1}$), Brugnerotto et al. [105] refer to the bands at $\tilde{\nu }=1320\;{cm}^{-1}$ as characteristic for *N*-acetylclucosamine and $\tilde{\nu }=1420\;{cm}^{-1}$ as reference band. This seems more suitable, as the spectral region around $\tilde{\nu }=3500\;{cm}^{-1}$ is strongly influenced by the presence of water [109]. All studies have shown that the evaluation of the absorbance is affected by the adjustment of the spectral baseline, indicating an influence of the person evaluating the spectra [109]. Additionally, scattering effects, moisture, or contaminations, such as proteins, showing comparable spectral features, may affect the reliability of the results. Nevertheless, IR spectroscopy is a valuable tool for estimating DA and DD, as analysis is quick and conveniently performed, and the technique evaluates entire samples, not only the soluble parts.

Besides titration-based methods [104,109,110,111,112] and viscosimetric experiments [104], 1H-NMR has been established as a benchmark method [109,113] for the determination of the DD.

Typical solvent systems consist of D_2_O [114] and acids, such as HCl [115], acetic acid, or their deuterated forms [116]. In-depth studies on relaxation times [115] and two-dimensional 1H-NMR [116] have also been published. Measurements at increased temperatures (T~343 K) and special pulse sequences were used to eliminate interference of the solvent peak HOD and account for the relaxation times of chitosan protons [117]. Validation of the 1H-NMR method in solution showed that the method is not only fast and precise but also reproducible, rugged, robust, and stable [117]. Experiments with 13C- and 15N-CP/MAS solid-state NMR show results, corroborating with 1H-NMR in liquid state [118,119].

A very powerful approach is based on ultraperformance liquid chromatography, coupled with evaporative light scattering and electrospray mass spectrometry for detection (UPLC-ELS-ESI-MS). Elaborated to study the enzymatic hydrolysis of chitosan hydrolases, this approach is not only suitable for the determination of the DD but also the investigation of the pattern of acetylation (P_A_) on chitosan oligomers. The method is described as accurate as 1H-NMR but requiring far less sample. Detection and evaluation can be performed label-free, but also with multiple isotopic labelling for quantification [120,121].

### 3.3. Determination of Molecular Weight

The molecular mass of chitin and chitosan is another critical parameter influencing biological and physicochemical properties [122]. Thus, accurate and reliable determination of this parameter is crucial for understanding and applying the biopolymers. Nowadays, gel permeation chromatography (GPC), also referred to as size exclusion chromatography (SEC), is most widely used.

Almost parallel to the development of chemical analysis, reports on the determination of the molecular weight of chitosan can be found in the literature. Early methods are based on colligative properties, such as the increase in the boiling point of a solution [123], the reduction of the vapor pressure [124], or measuring the osmotic pressure between differently concentrated solutions (Barger method) [125]. Another way to determine molecular weight is by investigating the sedimentation behavior in an ultracentrifuge according to the Archibald method, as described by Elias [126]. The sedimentation coefficient is derived from the concentration gradients. From this coefficient, the molecular weight can be calculated.

GPC, one of the most commonly used methods today, was first introduced on polysaccharides in 1976 by Basedow et al. The sample, which is prepared as a solution, is applied to a chromatographic column, and the elution volume of the sample, the exclusion volume, and the total volume of the column are determined. The molecular weight can be obtained by calibrating elution volumes of samples with standards of defined molecular weight. A close chemical and structural resemblance between the standard and the sample is important for an accurate estimation of the actual molecular mass of the sample. As no defined chitosan samples covering a large range of molecular mass is available, other, more or less representative, standards have been used.

Wu [127], Lavertu et al. [128], Yen et al. [129], and Chen and Hwa [130] describe methods using dextran as a calibration standard. Other standards used are D-glucans [131], pullulan [132], *N*-acetylchitooligosaccharides [133,134,135,136], or polyethyleneoxides [128].

To overcome the shortcomings of less representative standards, special detectors can be used for analyzing the fractions obtained after separation with GPC. Especially the combination of GPC with multiple angle laser light scattering (GPC-MAL(L)S) and viscosimetric detection proved useful.

Light scattering and viscosimetric measurements alone are also suitable methods for the determination of the molecular weight of polymers. In contrast to GPC and MALDI-TOF-MS (see below), where distributions of molecular weights can be obtained, only average values of the molecular weight are accessible with static light scattering (SLS) or viscosimetric measurements. Investigation of the viscosity of chitosan solutions allowed the determination of the Mark–Houwink–Sakurada parameters for universal calibration of the viscosity detector in GPC.

The intrinsic viscosity $\left[\eta \right]$ of a polymer solution is given as (Mark–Houwink–Sakurada equation)

$$\left[\eta \right]=KM_{\eta }^{\alpha }$$

Here, K and α are the Mark–Houwink–Sakurada parameters, where K is a constant specific for the combination of solvent and polymer and the exponent α describes the shape of the polymer in solution. $M_{\eta }$ is the viscosity weighed average of the molecular weight. Several authors have determined the parameters under different conditions (ionic strength, solvent) [110,137,138,139]. Investigations were mainly conducted on acidic aqueous systems in various types of viscosimeters [46,140,141,142,143].

A powerful yet demanding method for the investigation of molecular weights of polymers is matrix-assisted laser desorption/ionization time-of-flight mass spectrometry (MALDI-TOF-MS) [45,144,145,146,147]. Capable of investigating the molecular weight distribution of a sample, expensive apparatus and elaborate sample preparation are required. Typical matrices used for embedding chitosan for sample preparation are a-cyano-4-hydroxycinnamic acid [146] or 2,5-dihydrobenzoic acid [45,147]. Novel matrices, such as metallophthalocyanines, are also under investigation [145].

## 4. Application of Chitin and Chitosan Derived from Insects

After presenting analytic methods, the following section will be dedicated to the various applications for chitin and its derivative chitosan from insects, especially *Hermetia illucens*, or black soldier fly larvae. Due to the chemical similarity to chitin and its derivatives from crustaceans or fungi as a source, many further applications can be projected [25,148,149,150,151]. Although the use of BSFL-derived chitin and chitosan is not widely reported in the literature, some examples show the potential, especially considering the above-mentioned environmental benefits of insect farming. The possibilities range from packaging and water treatment to agriculture, pharmaceutical, and cosmetic applications (Figure 1) [152].

### 4.1. Packaging

Petroleum-based packaging poses enormous challenges for the world. The reason for this is the lack of biodegradability of plastics and the resulting environmental pollution [153]. For this reason, it makes sense to take a closer look at chitin as a non-toxic and natural resource that is biodegradable [154,155].

The study by Le et al. [156] describes an environmentally friendly method for the extraction of chitin and nano chitin from the black soldier fly using a co-solvent system of glycerol and hydrochloric acid. The extracted chitin fibrils have a width of 34 nm, a length of 494 nm, and a high crystallinity of 59.18%, indicating an effective removal of proteins and lipids. This chitin has been successfully incorporated into gelatin-based films to improve their antioxidant and physical properties, such as thickness, grammage, opacity, moisture content, and water solubility. Compared to the traditional method that produces α-chitin, the co-solvent method produces *γ*-chitin and is faster as well as more environmentally friendly. The developed chitin/gelatin films show great potential as antioxidant bio-packaging films, although optimizations are still needed to further improve their appearance and bioactivity.

Triunfo et al. [157] used chitosan derived from the pupal exuviae of the BSFL, which was successfully tested as a preservative coating for fresh apricots, nectarines, and peaches. It showed similar or better efficacy compared to commercial chitosan from crustaceans in maintaining the storage conditions of fruits. The use of chitosan from BSFL offers an environmentally friendly and sustainable alternative, as it is derived from insect farming waste products, supporting a zero-waste circular economy [157,158]. Chitosan-coated fruits showed a longer shelf life, both at room temperature and under refrigeration, as mold growth and weight loss were reduced. Future research could focus on optimizing chitosan concentrations and combining it with other natural active ingredients to further improve its preservation properties.

Moreover, chitosan was extracted from the pupal exuviae of BSFL and used for the preservation of fresh cherry tomatoes, showing comparable or prolonged freshness compared to commercial chitosan [159]. The studies showed that the application of chitosan by spraying was significantly more effective than the immersion method, especially in terms of reducing weight loss and stabilizing the pH of the tomatoes. Storage at 4 °C was found to be more beneficial for maintaining physicochemical stability, increasing the concentrations of phenols and flavonoids, and preserving antioxidant activity compared to storage at room temperature. Interestingly, heterogeneous chitosan showed higher efficacy in stabilizing physicochemical parameters such as pH and mass in the tomatoes, while homogeneous chitosan positively influenced the concentration of bioactive compounds and antioxidant activity. These findings highlight the potential for optimization of chitosan formulation, and further research is needed to maximize the efficiency and application of insect-derived chitosan for food safety and preservation.

Falgayrac et al. investigated the value creation of by-products of the black soldier fly through the extraction and processing of chitin and chitin nanocrystals (CTNCs) [11]. Chitin was extracted from the larval skins, pupal exuviae, and dead flies by a four-step chemical extraction, followed by isolation of CTNCs by acid hydrolysis. The yields varied between 5.8% and 20%, depending on the by-product. The isolated chitin nanocrystals exhibited a rod-like morphology with heights between 13 and 27 nm, making them potential nano-enhancing agents. These CTNCs offer versatile applications, especially in the development of nanocomposites and sustainable packaging materials, which are of interest in various industrial fields.

In their study, Pasquier et al. [24] investigate the properties of chitin nanofibers (CTNFs) isolated from *Hermetia illucens* and compare them with commercial chitin nanofibers obtained from shrimp shells. The results show that the CTNFs from *Hermetia illucens* are comparable to commercial counterparts in many respects, particularly in terms of their physicochemical properties, including chemical structure, thermal stability, and morphological characteristics. A significant improvement was observed in fibrillation, resulting in suspensions with higher light transmittance and films with increased transparency [12]. The mechanical properties of the films prepared from *Hermetia*-CTNFs were like those from shrimp-CTNFs, with the differences mainly due to the better fibrillation and homogeneity of the fibers from the insect source. These results indicate that chitin from *Hermetia illucens* is a promising and sustainable alternative to commercial chitin from crustaceans, especially in applications requiring high transparency and mechanical stability, such as packaging.

As promising materials for the packaging industry, chitin and chitosan are characterized by several key advantages [155]. In contrast to conventional plastics based on fossil raw materials, both biopolymers are completely biodegradable and therefore make a significant contribution to reducing plastic waste [155]. Particularly noteworthy are the antimicrobial properties of chitosan, which provide an effective protective barrier against microbiological contamination and can therefore extend the shelf life of food [155]. In addition, the extraction of chitin and chitosan from waste products, such as the exuviae of the black soldier fly, enables the sustainable and resource-saving utilization of organic by-products [155]. Due to their versatility, these biopolymers can be used in various forms, such as films and coatings, and offer excellent barrier properties against oxygen, oil, and moisture, which makes them particularly suitable for use in food packaging [155]. These properties underline the potential of chitin and chitosan as sustainable alternatives to conventional plastics in the packaging industry [155].

### 4.2. Agriculture

The study of Bulak et al. [160] shows that the puparia of the black soldier fly are suitable to produce biochar, which has a high nitrogen content and stimulates plant root growth. The physicochemical properties of the biochars, such as basic pH value, specific surface area, and pore structure, vary with the pyrolysis temperature. The biochar contains numerous micro and macro elements and has a moderate ability to adsorb heavy metals such as lead, cadmium, and nickel, which makes it useful for soil improvement. Ecotoxicological tests showed no toxicity to plants and soil invertebrates and even promoted root growth and reproduction. The use of chitin-rich insect waste for biochar production is an environmentally friendly and sustainable method of waste management in insect farming.

Kemboi et al. [161] showed that chitin and chitosan derived from the pupal exuviae of BSF are effective in inhibiting the growth of *Ralstonia solanacearum,* causing tomato wilt, a disease among the most destructive for tomato crops worldwide, leading to significant yield losses. The application of BSF-chitin and chitosan to the soil reduced disease incidence by 30.31% and 34.95%, respectively, and disease severity by 22.57% and 23.66%, respectively. These findings suggest that black soldier fly pupal exuviae serve as a valuable and renewable resource for chitin and chitosan, which show great promise as environmentally friendly antimicrobial agents against bacterial wilt in tomatoes.

### 4.3. Water Treatment and Absorption

Bazan-Wozniak et al. demonstrate the potential of *Hermetia illucens*-based materials for the efficient removal of pollutants in aqueous systems. The study investigates the production of porous activated carbon from the pupal exuviae of the black soldier fly for the efficient adsorption of methylene blue from aqueous solutions. By chemical activation with potassium carbonate, a particularly high surface area development of 1167 m^2^/g and an excellent adsorption capacity of 641 mg/g could be achieved. The adsorption mechanism is mainly based on chemisorption, whereby the pH value of the solution and the surface charge of the material play an important role. In addition, the carbon activated with potassium carbonate is highly reusable, as it can be regenerated and reused after adsorption.

Furthermore, it was shown that chitin can be isolated from black soldier fly exuviae with stable physicochemical properties, including high crystallinity (60%) and thermal stability (decomposition temperature of 392 °C) [162]. A key result is the effective adsorption of nickel ions enabled by ionic exchange and complex formation. The different extraction methods, especially the use of hydrogen peroxide, significantly improved the adsorption properties of chitin. These results underline the potential of chitin from *Hermetia illucens* for environmentally friendly applications, especially in the field of heavy metal removal from aqueous solutions, as well as possible industrial applications in the field of water treatment and wastewater treatment.

In the study from Elouali et al. [163], chitosan from black soldier fly pupal exuviae was successfully used to treat olive mill wastewater and showed high efficiency in the removal of heavy metals such as iron and copper, with elimination rates of up to 86.84% for iron and 82.14% for copper. Phosphorus was also removed at a rate of 43.47%. The best results were achieved under optimized conditions at 45 °C and intensive agitation, which increased the adsorption capacity of the chitosan. In addition, the content of polyphenols in wastewater was reduced by up to 77%, confirming the suitability of chitosan for organic pollutant removal. Overall, chitosan offers an environmentally friendly and effective alternative to conventional chemical coagulants in wastewater treatment.

Ben Amor et al. [164] explored the use of insect-derived chitosan for the removal of the dye methylene blue from wastewater. Chitosan was extracted from various insect species, including *Blaps lethifera*, *Pimelia fernandezlopezi*, and *Musca domestica.* The efficiency of dye removal strongly depends on the insect source of chitosan, ranging from 10.3% (*M. domestica*) to 87.7% (*B. lethifera*), showing the potential for wastewater treatment but also the need for further research to fully understand the underlying mechanisms. The study highlights that insects represent a sustainable source of chitosan due to their rapid reproduction, ease of cultivation, and high resilience to environmental changes, especially as the properties of the extracted materials are comparable to chitosan obtained from crustaceans.

### 4.4. Pharmaceuticals and Cosmetics

Polysaccharides distinguish themselves among other renewable polymers due to their abundance and versatility in biomedical and pharmaceutical applications, offering excellent biocompatibility and ease of chemical modification [165,166]. Among other possibilities for functionalization, their abundant functional groups enable crosslinking [167] for gel formation or hydrophobization for the formulation of nanoparticulate structures [168,169].

Chitosan obtained from different developmental stages of the black soldier fly shows significant antimicrobial activity against both Gram-negative bacteria such as *Escherichia coli* and Gram-positive bacteria such as *Micrococcus flavus* [170]. Compared to commercial chitosan from crustaceans, the chitosan from *Hermetia illucens* shows comparable or even better antimicrobial properties [170,171]. Both bleached and unbleached chitosan from larvae, pupal exuviae, and dead adults exhibited antimicrobial activity, with pupal exuviae providing the best results.

In their study, Khayrova et al. [171] investigated the antimicrobial effect of chitosan obtained from the larva of the black soldier fly in comparison to chitosan from crabs. About the antibacterial effect against *Staphylococcus epidermidis* (Gram-positive), chitosan from *Hermetia illucens* showed a stronger efficacy than crab chitosan. The minimum inhibitory concentration (MIC) for *Hermetia illucens* chitosan was 62.5 µg/mL, while crab chitosan required an MIC of 125 µg/mL. This indicates that black soldier fly chitosan is effective at lower concentrations and thus has higher antibacterial activity. Against *Escherichia coli* (Gram-negative), both chitosan species exhibited a weaker effect, with MIC values exceeding 500 µg/mL, illustrating the lower sensitivity of this bacterium to chitosan. Chitosan from *Hermetia illucens* also showed better results in antifungal activity compared to crab chitosan, especially against the fungus *Fusarium oxysporum*. At concentrations of 0.23 mg/mL and molecular weights (M_w_) of 19.64 kDa and 21.36 kDa, respectively, the metabolic activity (MA) of *F. oxysporum* decreased below 50%. In contrast, when crab chitosan was used at the same concentrations, the MA was 50% or higher, indicating lower antifungal activity [18]. Against *Botrytis cinerea*, metabolic activities remained ≥50% at both chitosan concentrations tested, indicating an overall lower efficacy against this fungus.

Lin et al. [172] show in their study that chitin and chitosan can be successfully extracted from the pupal exuviae of the black soldier fly by microbiological fermentation with *Bacillus licheniformis* A6. The recovery rate of chitin was about 12.4%, and the degree of deacetylation (DDA) of chitosan was about 81.5%, as determined by UV–vis spectroscopy. In terms of antimicrobial activity, BSF-chitosan showed strong bactericidal effects against *Pseudomonas aeruginosa* with a minimum inhibitory concentration (MIC) of 0.04 mg/mL and a minimum bactericidal concentration (MBC) of 0.16 mg/mL. Against *Staphylococcus aureus*, the MIC was 0.60 mg/mL, and the MBC was 1.25 mg/mL, indicating a higher concentration for inhibition and bactericidal activity compared to *P. aeruginosa*. The presented results support the applicability in pharmacy and cosmetics.

A further step in the application of BSF chitosan is shown by Alghuthaymi’s study [173], which investigated the antibacterial effect of chitosan/gum arabic nanocomposites containing eugenol and selenium nanoparticles (SeNPs). Chitosan was extracted from the larva of the black soldier fly and processed into nanoparticles (NCT) conjugated with gum arabic (GA). Eugenol and biosynthesized SeNPs were loaded into the NCT/GA nanocomposite. The resulting nanocomposites (NCT/GA, NCT/GA/Eug, and NCT/GA/Eug/SeNPs) showed a strong antibacterial effect against *Escherichia coli* and *Staphylococcus aureus*, which exceeded the effect of the standard antibiotic. The treated bacteria showed clear deformations after 5 h and were completely lysed after 10 h.

The combination of chitosan (Cs), propolis extract (Pro), and silver nanoparticles (Ag-NPs) also showed strong antimicrobial activities against skin pathogens such as *Staphylococcus aureus* and *Candida albicans* and promoted rapid wound healing in rats [174]. The silver nanoparticles were successfully synthesized using propolis extract by an environmentally friendly biogenic method, resulting in spherical nanoparticles with an average diameter of 8.73 nm. Cs was extracted from black soldier fly larvae with a yield of 1.56%, a degree of deacetylation of 91.3%, and a molecular weight of 88600 Da. The antimicrobial activity of the Cs/Pro/Ag-NPs combination surpassed that of commercial antibiotics such as vancomycin and nystatin, highlighting its potential as an alternative antimicrobial agent. In addition, treatment of rat wounds with this combination resulted in faster healing without signs of inflammation or infection, making it particularly suitable for skin protection, disinfection, and wound regeneration applications.

Another possible application is hydrogels made from chitosan. Current publications either do not specify a chitosan source or use crustaceans as raw material. An analogous transfer from crustacean chitosan to insect chitosan is to be expected due to the same chemical structure. However, the above-mentioned differences in the preparation of chitosan from the different species have to be considered.

Flores-Espinoza et al. [175] investigate the properties of gelatin-chitosan hydrogels for dental applications. Chitosan is incorporated into the hydrogels due to its bioadhesive, biocompatible, and antibacterial properties. The hydrogels were synthesized with chitosan concentrations of 0%, 0.2%, and 0.5% and characterized using FTIR spectroscopy. The hydrogels exhibited varying degradation rates under hydrolytic and enzymatic conditions, showing that higher chitosan concentrations lead to prolonged degradation times. Additionally, chitosan-containing hydrogels demonstrated a slower but more controlled swelling capacity compared to pure gelatin hydrogels. In vitro cytotoxicity tests revealed that the 0.2% chitosan hydrogels exhibited no cytotoxic effects, whereas the 0.5% hydrogels showed moderate cytotoxicity. Antibacterial activity against *Streptococcus mutans* was significantly enhanced with increasing chitosan concentrations. Furthermore, the mechanical properties of the hydrogels improved in the presence of chitosan, leading to increased stability. In summary, gelatin-chitosan hydrogels exhibit promising characteristics for postoperative dental care as they offer controlled degradability, enhanced mechanical stability, supportive effects on cell proliferation, and strong antimicrobial properties, making them potential candidates for bioactive wound dressings or drug delivery carriers in dentistry.

Ma et al. [176] describe the development of a thermo- and pH-sensitive hydroxypropylchitin hydrogel with tannic acid and iron ions (Fe^3+^) for wound healing. The hydrogel can be applied as a liquid and forms a gel matrix at body temperature that fills irregular wound surfaces. Tannic acid improves the mechanical properties, has an antibacterial effect, and is released in the acidic environment of infected wounds in a pH-dependent manner. The hydrogel shows broad antibacterial activity against *E. coli* and *S. aureus*, promotes cell migration of fibroblasts, and accelerates wound healing in animal studies, including scar-free regeneration with skin appendages such as hair follicles. It is biodegradable, durable, and offers potential as an injectable, antibacterial wound dressing.

Chitosan and chitosan oligosaccharides have garnered significant attention for their potential in regulating blood lipids and sugar levels. Studies have demonstrated that chitosan oligosaccharides effectively reduce serum cholesterol by enhancing cholesterol transport to the liver and promoting its excretion [177]. Additionally, chitosan oligosaccharides increase the expression of low-density lipoprotein receptors, facilitating lipid accumulation in cells and lowering blood lipid levels [178]. In terms of blood sugar regulation, chitosan combined with metformin has shown synergistic effects, improving drug efficacy and reducing overdose risks in diabetic models [179]. Furthermore, chitosan oligosaccharides enhance insulin sensitivity and glucose control, partly through gut microbiota modulation [180]. These properties position chitosan and its derivatives as promising agents for managing hyperlipidemia and diabetes. The combination of chitosan oligosaccharide ingestion and exercise, such as running, improved the immune system by increasing the spleen-to-body weight and lung-to-body weight ratios in Sprague-Dawley rats [181]. Chitosan oligosaccharides have been used as feed additives in animal nutrition to promote growth performance, reduce diarrhea, and improve nutrient digestibility, which also indirectly supports immune system health [182,183].

The study by Omer et al. [184] describes the development of a pH-sensitive hydrogel based on aminated chitosan (AmCs) and gelatin (gel) for oral drug delivery. The hydrogel showed pH-dependent swelling profiles: a higher AmCs content increased swelling at pH 1.2 and reduced it at pH 7.4, while a higher gelatin content increased the loading efficiency up to 79%. Drug release was studied over 6 h in simulated gastric and intestinal fluids and showed controlled release in the stomach and reduced release in the intestine. Biocompatibility tests confirmed the biodegradability and lack of toxicity of the hydrogel. Overall, the AmCs gel hydrogel enables targeted and controlled drug release in the gastrointestinal tract, which improves bioavailability, reduces side effects, and supports more efficient drug delivery.

Due to its antioxidant, moisturizing, antimicrobial, and biocompatible properties, chitosan is highly valued in cosmetics. It serves as an active ingredient and carrier for other active ingredients. Possible applications of chitosan-based materials in cosmetic applications are comprehensively reviewed by Kulka and Sinokowska [185]. Chitosan is characterized by its high water-binding capacity, which effectively enhances skin hydration [186,187]. By forming a hydrophilic film on the skin, it reduces transepidermal water loss and helps maintain moisture levels. These properties make chitosan a valuable ingredient in moisturizers and other skincare products. In addition, chitosan exhibits strong antimicrobial properties due to its polycationic nature. The protonated amino groups interact with negatively charged structures on the surface of pathogens, leading to membrane destabilization and ultimately cell destruction [188]. This antimicrobial activity allows for a reduction in synthetic preservatives in cosmetic formulations, making chitosan an ideal ingredient for deodorants, antiperspirants, and anti-acne products [189,190]. Another advantage of chitosan is its ability to support wound healing. Through electrostatic interactions with negatively charged platelets and erythrocytes, it promotes blood clotting and accelerates tissue regeneration. In cosmetic applications, this property is utilized in regenerating face masks, which function similarly to wound dressings by supporting skin renewal [191,192,193]. Beyond its functional benefits, chitosan is biocompatible, biodegradable, and exhibits low cytotoxicity. It soothes the skin, has anti-inflammatory effects, and is particularly suitable for sensitive skin types as well as products designed to alleviate allergic reactions.

Chitosan gel nanoparticles (CNPs) improve the efficacy of skin care products by releasing the active ingredients in a targeted and controlled manner. Applications include anti-aging products, moisturizers, sunscreens, and acne treatments [194,195]. Chitosan derivatives such as carboxymethylchitosan offer additional benefits such as improved moisture absorption and antioxidant properties. The versatility and sustainability of chitosan from BSFL make it a promising ingredient in the cosmetics industry.

Melanin is a highly efficient natural photoprotector and radioprotector known for its ability to protect the skin from the harmful effects of UV and visible radiation, as about 99% of the absorbed radiative energy decays by non-radiative processes [31,196]. In a recent study, melanin was investigated in combination with chitosan to further increase its biological activity. A technology was developed to obtain natural conjugates from *Hermetia illucens*, which have the potential to combine the protective and bioactive properties of both substances [31]. By conjugating melanin with chitosan, the antimicrobial and antioxidant efficacy of chitosan could be further enhanced, opening interesting application possibilities in the development of cosmetic products such as sunscreens [31,196].

In a further study by Khayrova et al. [197], the chitosan-melanin complex (CS-M) was isolated from *Hermetia illucens* and processed into nanofibers, which have potential applications in wound healing. The melanin content and the resulting UV protection and antioxidant properties also open possibilities in tissue engineering. The molecular weight of the CS-M was 566 kDa and had a melanin content of 14%. The viscosity of the CS-M solutions was higher than that of pure chitosan, and gel formation was slower. Nanofibers with a diameter of about 250 nm were produced by electrospinning in combination with polyvinyl alcohol (PVA), which are water insoluble in the pH range of 3 to 8 and thus represent a sustainable source for wound dressings.

## 5. Conclusions and Future Perspectives

Chitosan, which is extracted from insects, shows enormous potential for applications in biomedicine, such as wound healing, tissue engineering, drug delivery, and antimicrobial therapies, because of its antioxidant, antibacterial, and anticarcinogenic properties.

Moreover, chitosan has shown biocompatibility, and it is eliminated from organisms, as it is water soluble. The abundance of functional groups allows chemical modifications to be easily introduced for creating and fine-tuning the properties of the functional material.

A major advantage of insects as a source of chitosan is their sustainability and environmental friendliness compared to conventional sources such as crustaceans.

There is also a lower risk of allergies when using insect chitosan, making it a safer alternative. As insect farming is still an emerging technology, future research and development has to focus on optimizing large-scale production to further advance commercial use. In addition, regulatory standards need to be developed to ensure the safety and efficacy of these novel materials.

Most of the applications based on crustacean- or fungi-derived chitosan are also accessible by insect-derived chitosan, as the chemical and physicochemical properties are comparable. Besides the environmentally beneficial production methods, there are also notable distinctivenesses, such as the natural occurrence of melanin in association with chitin in insects, opening up new avenues for research and innovative applications, especially in the field of cosmetics or pharmaceutics.

## Data Availability

No new data were created or analyzed in this study.
